# Raman Scattering Study of Amino Acids Adsorbed on a Silver Nanoisland Film

**DOI:** 10.3390/s22145455

**Published:** 2022-07-21

**Authors:** Alexey Skvortsov, Ekaterina Babich, Andrey Lipovskii, Alexey Redkov, Guang Yang, Valentina Zhurikhina

**Affiliations:** 1 Institute of Biomedical Systems and Biotechnology, Peter the Great St. Petersburg Polytechnic University, Polytechnicheskaya 29, 195251 St. Petersburg, Russia; colbug@mail.ru; 2 Laboratory of the Molecular Biology of Stem Cells, Institute of Cytology, Russian Academy of Sciences, Tikhoretsky 4, 194064 St. Petersburg, Russia; 3 Institute of Physics and Mechanics, Peter the Great St. Petersburg Polytechnic University, Polytechnicheskaya 29, 195251 St. Petersburg, Russia; babich_es@spbstu.ru (E.B.); zhurihina_vv@spbstu.ru (V.Z.); 4 Laboratory of Nanophotonics, Alferov University, Khlopina 8/3, 194021 St. Petersburg, Russia; 5 Department of Physics and Technology of Nanostructures, Alferov University, Khlopina 8/3, 194021 St. Petersburg, Russia; 6 Institute for Problems in Mechanical Engineering of the Russian Academy of Sciences, Boljshoy Prospekt V.O. 61, 199178 St. Petersburg, Russia; red-alex@mail.ru; 7 School of Materials Science and Engineering, Shanghai University, Shangda Rd. 99, Baoshan, Shanghai 200444, China; guangyang@shu.edu.cn

**Keywords:** SERS, metal surfaces, amino acids

## Abstract

We studied the surface-enhanced Raman spectra of amino acids *D*-alanine and *DL*-serine and their mixture on silver nanoisland films (SNF) immersed in phosphate-buffered saline (PBS) solution at millimolar amino acid concentrations. It is shown that the spectra from the amino acid solutions differ from the reference spectra for microcrystallites due to the electrostatic orientation of amino acid zwitterions by the metal nanoisland film. Moreover, non-additive peaks are observed in the spectrum of the mixture of amino acids adsorbed on SNF, which means that intermolecular interactions between adsorbed amino acids are very significant. The results indicate the need for a thorough analysis of the Raman spectra from amino acid solutions, particularly, in PBS, in the presence of a nanostructured silver surface, and may also be of interest for studying molecular properties and intermolecular interactions.

## 1. Introduction

The surface-enhanced Raman scattering (SERS) of organic molecules by the surface plasmon resonance (SPR) of metallic structures and surfaces is a promising sensing technique that has gained considerable interest in recent decades [1]. Metal particles are often used in SERS as they are more expendable, easy to produce, and can provide larger enhancement than bulk metal [2]. There is a large amount of data on the excellent sensitivity and temporal resolution of SPR biosensors, which allows the detection of single molecules [3], runtime measurements [4], microscopic hyperspectral imaging [5], among others. However, surface chemistry plays a very important role in the formation of SERS spectra. In general, patterns and regularities observed in a bulk solution Raman spectra cannot be extrapolated to SERS spectra of an analyte adsorbed by a metal surface [6,7,8], and thorough studies are required. The multiplexed sensing of several analytes by SERS also raises questions about differences in analyte adsorption on a metallic surface and the interaction of different analytes at the surface. Of considerable interest is the effect of inorganic ions on the analyzed solutions. Most of the reference SERS spectra of organic molecules are measured in ultrapure water solutions. However, in potential biosensing applications, the target molecules are usually isolated in solutions containing various inorganic ions, and the removal of these ions is costly and sometimes undesirable, as it may ruin the native structure of biomolecules. The presence of inorganic ions may affect not only the structure of target molecules but also modify the physicochemical properties of the metallic surface, especially for less noble metals.

Natural amino acids are important test objects in SERS studies, as they possess various chemical groups characteristic of biomolecules and are important analytes *per se*. A considerable amount of data on the SERS studies of amino acids with various metallic enhancing substrates have been obtained [9,10,11]. The SERS of amino acids has been used both as a standalone analytical method and as part of hyphenated techniques [12].

Different metal substrates can be used to produce SERS spectra, including roughened surfaces and various nanoparticulate states of silver [10,13,14]. One of the promising SERS substrates for biosensing applications are silver nanoisland films (SNF), which can be produced by the out-diffusion of metal from glass substrates [15]. It has been previously shown that SNF could enhance the Raman spectra of various molecules [16,17,18,19]. We have recently shown that SNFs formed by out-diffusion are not inherently stable in phosphate buffer saline solution (PBS), which is widely used in biochemical and cell studies, and other buffer systems [20]. The film gradually reacts with the medium and releases silver ions into the solution, but its ability to enhance Raman scattering is retained. However, it was noted that the enhanced spectra of *D*-alanine (Ala) dissolved in various buffers differed from each other and from the spectrum of amino acid crystallites [21].

The present work investigates the surface-enhanced Raman spectra of amino acids *D*-alanine (enantiopure) and *DL*-serine (Ser; racemic) and their mixture on SNF immersed in standard PBS solutions at 1 mM amino acid concentrations.

## 2. Materials and Methods

**Figure 1:**
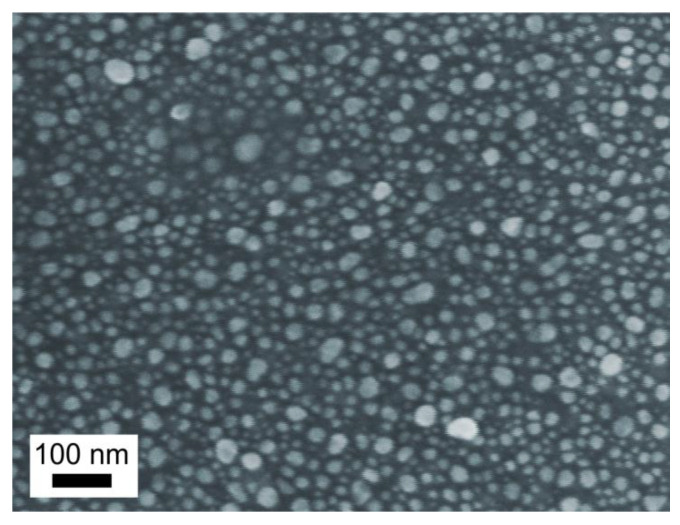
SEM image of the prepared SNF.

SNFs, which are ensembles of silver nanoparticles randomly distributed over a glass surface, were prepared by doping a soda-lime glass slide (Agar Scientific Ltd., Essex, UK) with silver ions via silver-to-sodium ion-exchange and annealing the silver-containing glass in a hydrogen atmosphere, as described elsewhere [16,20]. For ion-exchange, we immersed the slide for 20 min in AgNO_3_-NaNO_3_ melt containing 5 wt.% of AgNO_3_ (LenReactiv, St. Petersburg, Russia) heated to 325 °C. Annealing in hydrogen was performed for 15 min at 250 °C. A typical scanning electron microscope (SEM, Supra 25, Zeiss, Oberkochen, Germany) image of the prepared SNF is demonstrated in Figure 1.

Ala and Ser (pure grade, Reakhim, Moscow, Russia) in the form of both powders (crystallites) and 1 mM solutions in PBS, pH 7.4 (Biolot, St. Petersburg, Russia), were studied using a confocal Raman microscope (Alpha 300R, Witec, Germany) equipped with a 532 nm excitation laser (output power of ~30 mW) and a 10 ×/ 0.25 objective (excitation spot diameter ~3 μm). The spectra were collected from the crystallites placed on the SNF or glass surface and from the SNF/glass surface during their immersion in the analyte solutions. In the last case, the glass slides with/without SNF were placed face up in a cuvette filled up with ~5 mL of the solution at ambient temperature (~20 °C), and the spectra were collected in the close vicinity of SNF/glass surface in different positions on the sample’s surface (~300 spectra). Each measured spectrum corresponded to an accumulation time of 1 s.

For peak detection, each spectrum of the data matrix, obtained by scanning the sample surface, was subjected to Savitzky–Golay smoothing, and the baseline was removed using the rolling ball algorithm. The peak positions were calculated using an OriginLab^®^ implementation of the local maximum method [22]. The peaks whose positions differed by less than ±5 cm^−1^ were considered as one peak with the position of the most pronounced peak. For peak intensity analysis, cosmic spikes and baseline were removed from each spectrum using the PALS algorithm with a smoothness parameter of 10^5^ [23] and then subjected to standard principal component analysis (PCA). Outlier spectra (1–3 spectra per dataset, presumably corresponding to SNF defects) were assessed manually and omitted from further analysis.

## 3. Results and Discussion

The Raman spectra of the Ala and Ser crystallites placed on SNF coincided with their spectra on glass without SNF; the spectra also completely matched the respective Ala and Ser reference spectra presented in the literature [24]. At the same time, SNF immersed in PBS solutions of Ala and Ser showed spectra that were different from the ones obtained for crystallites, and no measurable signal was obtained when a glass without silver nanoislands was immersed in PBS solutions of amino acids. This firmly indicated that, for the case of data collection from a PBS solution, the observed Raman signal is produced by amino acid molecules adsorbed on SNF.

The similarity of the spectra of the crystallites placed on glass and SNF is because the contribution to Raman scattering from molecules in the bulk of amino acids crystallites, which are not bound to SNF, dominates over the Raman signal from several layers of molecules bound to SNF. Indeed, the SPR of the metal nanoisland can only “interact” with the molecules at a distance of no further than half of the radius of the nanoisland [25]; in our case, this is less than 5 nm (see Figure 1), while the total number of molecules that contribute to Raman scattering is proportional to the depth of focus, which is ~100 μm. The situation is different for the experiments with PBS solutions. The drastic decrease in the density of the molecules from ~10^28^ (in crystallite) to ~10^23^ (in PBS solution) per m^3^ results in a sufficient weakening of the Raman scattering, and no measurable signal was obtained from glass without SNF or the same from a PBS solution. Thus, when glass with SNF is immersed in PBS solutions, the contribution to Raman scattering from the molecules that “interact” with SPR of nanoislands (bound molecules) is dominant. This is because the “interaction” results in a several-orders-of-magnitude enhancement of Raman scattering (up to ~10^4^ [20]).

**Figure 2:**
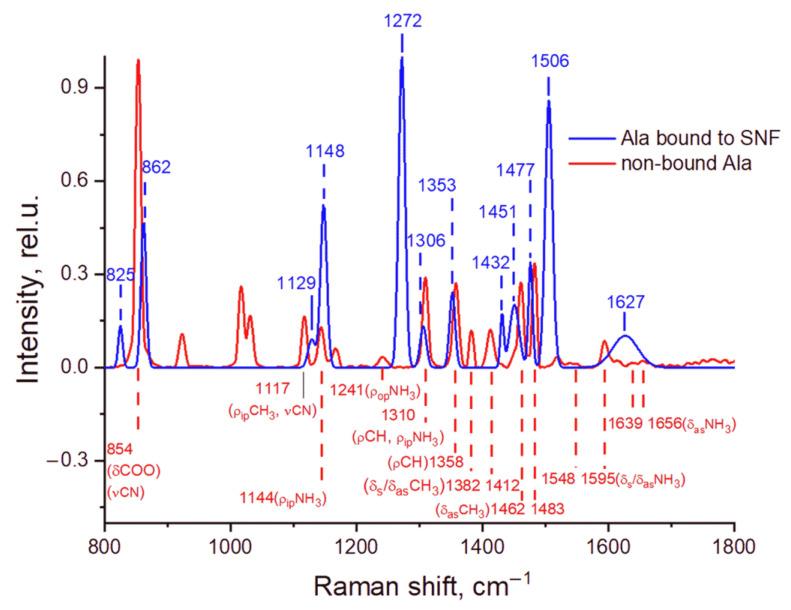
Comparison of Raman spectra of Ala bound to SNF (blue) and non-bound (crystallite) Ala (red). The peaks of crystallite spectrum are assigned to vibration modes of C−N, NH_3_, and CH_3_ units: ν-stretching, δ-scissoring, ρ-rocking (ip—in plane, op—out of plane) [26]. The presented refined spectra were obtained by fitting the Gaussian peaks to the respective averaged spectra.

The comparison of the averaged Raman scattering spectra of the non-bound Ala and Ala bound to SNF is presented in Figure 2. The peaks in the spectrum of non-bound Ala are assigned to vibration modes of Ala crystallites in accordance with reference [26]. One can see in Figure 2 that the spectrum of Ala bound to SNF differs from the reference spectra of Ala crystallites. This difference is much more profound than the difference between the spectra of crystallites and the published spectra of concentrated aqueous solutions (these spectra are relatively similar for alanine) [24]. Most peaks assigned to vibration modes of C-N, NH_3_, and CH_3_ shifted, while the peaks at 923 and 1017 cm^−1^ assigned to the vibration modes of COO [27] and C-C are missing. Due to strong shifts, the assignment is not unambiguous, but it clearly indicates that the binding mostly leads to a shift and an enhancement of the peaks corresponding to NH_3_. On the contrary, in the SERS spectra, there are no peaks corresponding to COO. This observation is different for several reports of SERS spectra of alanine on a silver surface in distilled water, in which most peaks can be mapped to peaks of crystallite alanine and reliably assigned. In distilled water, the changes in peak intensities usually evidence the binding of amino acid to metal through the COO group [9,10] with a very weak enhancement of the NH_3_ group, but in our conditions, this mode of binding is not supported by the observed spectrum. It should be noted that in some studies of amino acids, an enhancement of the NH_3_ group has also been observed [11], so the mode of binding is not universal.

**Figure 3:**
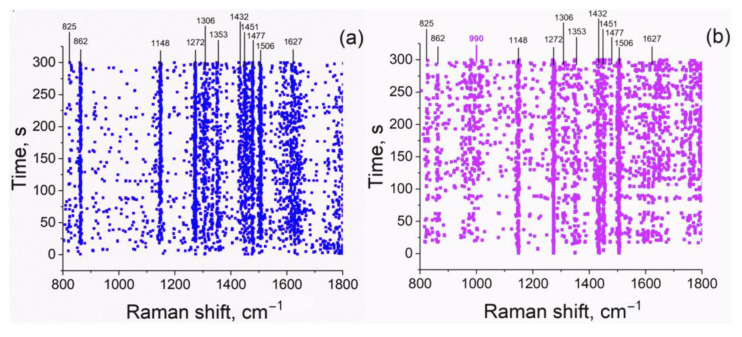
Raman peaks positions maps for (**a**) Ala and (**b**) Ser bound to SNF in PBS. Each point on the map corresponds to a feasible peak in the respective spectrum, as detected by the local maximum method [22].

To evaluate the stability and uniformity of the signal of the Ala molecules adsorbed on SNF, we scanned the SNF surface with a time step of 1 s. The spectrum appeared immediately after the SNF was immersed in Ala solution and did not change with time. The signal-to-noise ratio of a single run was moderate, especially if compared to a dry sample, but the individual Raman peaks of the bound Ala were clearly visible and firmly detected (Figure 3a). The first principal component (PC1) of the data matrix corresponded to ~25% of the total variance; its loading matched the average spectrum, while the loadings of higher order PCs had no spectral features. Thus, there was only one significant spectrum, which was observed from all points of the sample, and no dependence on time or position was detected. So, we can conclude that *D*-alanine binds to SNF rather rapidly, no slow change takes place, and only one mode of binding could be observed (the latter, however, may be an average of fast processes).

**Figure 4:**
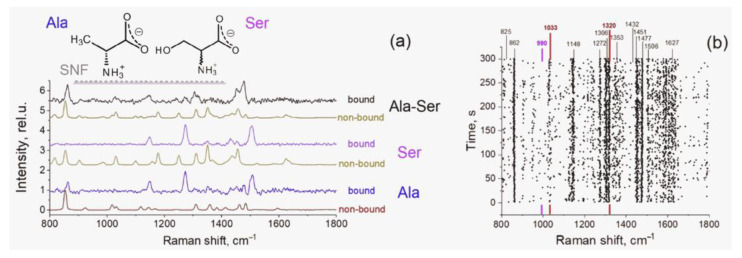
(**a**) Most significant spectral contributions (PC1 loadings) in the data matrices of Raman spectra of Ala, Ser, and 1:1 Ala-Ser mixture (theoretical sum) bound to SNF in PBS and in crystalline form (non-bound) Top: chemical structures of Ala and Ser in the vicinity of SNF (below the structures). (**b**) Raman peaks positions map for Ala-Ser mixture bound to SNF in PBS: black labels—Ala and Ser common peaks, magenta label—Ser only peak, red labels—Ala-Ser only peaks.

The evaluation of Ser bound to SNF in PBS produces similar results (Figure 3b). Except for the 800–1000 cm^−1^ region, the spectra of bound Ala and Ser are quite similar. In fact, they are more similar to each other in terms of peak positions and relative intensities than to the spectra of the respective crystalline states (see Figure 4a). The Ser spectrum has only one additional weak peak at 990 cm^−1^, which can be attributed to C-N stretching [28]. The first principal component corresponds to ~60% of the total variance of the data matrix, and the other components are effectively noise. The principal spectrum of Ser bound to SNF is apparently simpler than the spectrum of bound Ala, despite the fact that Ala is chemically simpler. This may be a result of peak splitting and their disappearance in noise; alternatively, it may be the result of some kind of additional ordering by intermolecular interaction (see below). The overall Raman signal for bound Ser was stronger than for bound Ala, but it displayed large irregular point-to-point variations (up to 10-fold changes in PC1 scores were observed).

These observations indicate that the binding of Ala and Ser molecules to silver surfaces is structurally similar and highly polarized. Due to the presence of 0.13 M of chloride in PBS, which strips part of silver as Ag(I) into chlorocomplexes in the solution, silver nanoislands in PBS contain excess electrons and gain an effective negative charge. As has been shown previously, e.g., in [7], the effective charge of the particles and the respective polarity of the double layer are very important for the preferred orientation of adsorbed organic molecules to the Raman-enhancing metal surface. At pH 7.4, which can be assumed to be constant because of the large buffer capacity of PBS, Ala or Ser molecules exist predominantly in a neutral zwitterionic state, similar to a crystal state or concentrated solutions. So, it is highly feasible that amino acid zwitterions orient themselves with positively charged NH_3_ group towards the surface and negative COO group away from it (which is dissimilar to silver surface in pure water solutions or surface-dried samples). Because of this, the peaks associated with NH_3_ group vibrations are most strongly affected by the local plasmons of the metal surface, while the other vibrations have an unfavorable orientation and/or are insufficiently enhanced. The potential protonation of the carboxylate group on binding (which is highly improbable at pH 7.4 but cannot be ruled out) does not interfere with the assumed orientation. As Ala and Ser are simple and differ only by a single group, the similarity of binding and enhancements is very tenable. Analogous effects were observed for other amino acids, which hints at a strong electrostatic orientation of the α-aminocarboxylic group by a silver surface [29]. It should be noted that the presence of chloride, which decreases the electrochemical potential of silver nanoparticles (standard redox potential for silver E°′ ~0.25 V in PBS), is important for tight binding. When SNF was immersed in distilled water solutions with neutral pH (E°′ ~0.8 V), no Raman peaks were observed even with 0.1 M of Ala and Ser (data not shown). Considering that the pK_a_ values of the studied amino acids are far from the used pH range, the charge state of amino acid molecules in PBS and distilled water are similar (zwitterion), so the difference should arise mostly from the change in the surface properties.

To test the ability to detect both amino acids in the mixture, we examined the Raman spectra collected in the vicinity of SNF immersed in the solution, containing 1 mM Ala and 1 mM Ser in PBS. The relative intensities of the Raman signals can be seen from the principal spectra in Figure 4a. The peaks positions map is shown in Figure 4b. Although the spectrum of the mixture on SNF and the spectra of the individual amino acids (bound and non-bound) have some common peaks and features, the response is far from additive, and a significant redistribution of intensities and the appearance of new peaks can be seen (Figure 4a). The spectrum of the mixture is also time- and position-independent, see Figure 4b (PC1 corresponded to 45% of total variance; PC2 comprised less than 2%, and the PC2 “spectrum” did not contain any characteristic Raman peaks). The total signal-to-noise ratio and point-to-point variations in the spectrum of the mixture are smaller than those in the spectra of bound Ser and similar to those of bound Ala.

Summarizing these data, we can conclude that serine and alanine molecules adsorb on silver nanoislands simultaneously with comparable affinity, forming a hybrid molecular layer. This layer is different from layers formed by pure amino acids and has a significant contribution from Ala–Ser complexes resulting in a specific pattern of peak intensities. The latter indicates significant interactions between adsorbed amino acids, which means that the layer of amino acid molecules on silver particles in the studied conditions is dense. Thus, non-covalent intermolecular interactions can make structural changes to the layers of oriented amino acid molecules on the silver surface, and these changes are strong enough to manifest in the positions and intensities of enhanced Raman peaks. This is quite dissimilar to solution studies, as specific non-covalent interaction of amino acids in the bulk aqueous media at the studied concentrations is negligible, and the spectral responses are generally additive [30]. While this finding is not beneficial to quantitative analytical applications, as it makes the calibration and rational spectral analysis more complicated, it is highly interesting for the studies of non-covalent intermolecular interactions of organic molecules in thin layers in an aqueous environment.

## 4. Conclusions

We have shown that the enhanced Raman spectra of *D*-alanine and *DL*-serine can be reliably observed using Raman microscopy from silver nanoisland films immersed in standard PBS solutions with millimolar concentrations of the amino acids. The obtained spectra significantly differ in peak intensities and positions from the Raman spectra of bulk solutions and crystallites of the corresponding amino acids on SNF and on glass. The effect is tentatively attributed to the electrostatic orientation of amino acid zwitterions by silver nanoparticles, which acquire a negative charge because of Ag(I) stripping by chloride in the solution, as the enhancement vanished if distilled H_2_O was used instead of PBS. The spectrum from SNF in amino acids mixture is not additive, which indicates a strong interaction of alanine and serine molecules on the SNF surface. While the spectra can be used to roughly discriminate pure amino acids and mixture, multivariate calibration would not be easy because the generalized Beer′s law is not satisfied for the mixture.

On the other hand, the observed effects may be used to study molecular properties and intermolecular interactions at a silver nanoparticle surface, which are negligible or too transient in the bulk solution. It is also important that the effective charge of SNF can be adjusted by changing the concentration of Ag(I)-stabilizing ligands (e.g., chloride) in the solution, providing an additional controlled parameter.
